# Lateral full-endoscopic lumbosacral foraminotomy for foraminal stenosis in dogs: technique development and initial case series

**DOI:** 10.3389/fvets.2026.1787235

**Published:** 2026-04-07

**Authors:** Colin J. Driver, Kerrie Morrison, Michaela Sojak, Jeremy Rose

**Affiliations:** Lumbry Park Veterinary Specialists, CVS Referrals, Alton, United Kingdom

**Keywords:** degenerative lumbosacral stenosis, endoscopic surgery, lumbosacral foraminotomy, minimally invasive surgery, veterinary spinal surgery

## Abstract

**Introduction:**

Degenerative lumbosacral stenosis (DLSS) is a common cause of pain in dogs, with lumbosacral foraminal stenosis (LSFS) frequently contributing to clinical signs through spinal nerve root compression. Conventional surgical approaches for foraminal decompression can be invasive and technically challenging. This study aimed to develop a lateral full-endoscopic lumbosacral foraminotomy (LELF) technique and evaluate its feasibility, safety, and effectiveness in dogs.

**Methods:**

LELF was first established in two large-breed dog cadavers, followed by clinical assessment in three dogs diagnosed with LSFS with cross-sectional imaging. Pre- and post-operative assessments included standardized DLSS scoring, owner-reported pain inventory (CBPI), and CT-based neuroforaminal volume measurements. Intra-operative parameters including incision length, surgical time, and visualization quality were recorded.

**Results:**

LELF was successfully performed on nine foramina (four cadaveric, five clinical) without conversion to open surgery. Incision length was < 10 mm, median surgical time was 95 min per foramen. All key anatomical landmarks were visualized, and no iatrogenic nerve root injuries occurred. Post-operative CT demonstrated significant enlargement of the lumbosacral foramina (median pre-operative volume: 315.2 mm3; post-operative: 605.6 mm3; *P* = 0.004). Clinical outcomes improved markedly at 6-month follow-up, with DLSS and CBPI scores indicating substantial pain reduction and restoration of excellent quality of life.

**Conclusion:**

LELF is a feasible and minimally invasive technique for LSFS in dogs, achieving effective foraminal decompression and favorable medium-term clinical outcomes. Further studies are warranted to assess long-term efficacy and to compare LELF with open and bi-portal endoscopic approaches.

## 1 Introduction

Degenerative lumbosacral stenosis (DLSS) is a complex spinal disorder caused by multiple pathological factors resulting in proliferative changes to the bone and soft tissues that compress the nerve roots of the cauda equina. It is the most common cause of lower back pain in medium- to large-breed dogs, but the clinical presentation of DLSS varies considerably and partly depends on which nerve roots are affected by the pathology (1). Nerve root compression has been identified as the strongest predictor of lumbosacral pain in a multivariable analysis of MRI changes, which is particularly relevant given the frequent coexistence of multiple pathological changes in dogs with DLSS (2). Lumbosacral foraminal stenosis (LSFS), involving compression of one or both exiting L7 spinal nerve roots within the L7–S1 intervertebral foramen, is present in 68% of dog with DLSS (3). LSFS causes radicular pain, pelvic limb lameness and/or radiculopathy (4). The intervertebral foramen starts in the lateral recess of the vertebral canal of L7 and extends in a curvilinear fashion caudal and ventrolateral to the pedicle; it can be divided into entry, middle and exit zones (5).

Surgical decompression of the L7 spinal nerve root should be considered if conservative therapy is unsuccessful (6). Dorsal approaches facilitate laminectomy of L7–S1, which only gives limited access to the foraminal middle- and exit-zones (7), as preservation of the articular facet joint is preferable to avoid instability (8). Conversely, lateral foraminotomy (9) decompresses the middle and exit zones but requires a dorsolateral approach to avoid the iliac wing, which limits visualization of the exiting nerve root and lead to large incisions with extensive muscle dissection.

In human medicine, minimally invasive endoscopic spinal surgery is expected to reduce peri- and post-operative morbidity, blood loss and hospitalization stay, and facilitate faster rehabilitation in comparison to open procedures (9–11). Endoscopic spinal surgery has also emerged in veterinary medicine as a tool to achieve the objectives of open procedures, such as pediculectomy (12), mini-hemilaminectomy (13), and limited-dorsal laminectomy (14). The feasibility of a bi-portal technique for lateral endoscopic lumbosacral foraminotomy has been described in dog cadavers (15). A uni-portal endoscopic approach to the spine involves a single skin incision for the introduction of a spine-specific, working channel endoscope. In humans, both bi- and uni-portal techniques have been successfully utilized to decompress neural structures with similar complication rates (16), although a single portal incision is inherently less invasive (17). The term “full endoscopic” has been described for procedures performed with a working channel endoscope (18). This distinguishes full-endoscopic procedures from “endoscope assisted” operations where tools are passed through trajectories separate from the endoscope, including bi-portal techniques.

The aim of this study was to develop a lateral full-endoscopic lumbosacral foraminotomy (LELF) technique in dog cadavers and to report its use in three dogs clinically affected by LSFS. We hypothesized that LELF would be feasible, safe, and effectively enlarge the L7–S1 foramen.

## 2 Materials and methods

### 2.1 Study population

The *ex-vivo* portion of the study consisted of two dog cadavers weighing >25 kg, euthanised for reasons unrelated to spinal disease and with no previous clinical history suggestive of spinal disease. Informed owner consent for scientific use of the cadavers was obtained.

Adult medium or large-breed dogs were recruited for the *in-vivo* clinical case series between January and March of 2025. The dogs were recruited if their histories were compatible with DLSS (including difficulty standing or jumping, episodic lameness, lower back pain on palpation, and an absence of orthopedic pathology identified on clinical examination), if they had undergone unremarkable radiologic assessment of the pelvic limbs within the preceding 3 months, and if a diagnosis of LSFS had been confirmed with magnetic resonance imaging (MRI) and computed tomography (CT) of the lumbosacral spine as previously described (1, 3).

### 2.2 Pre-operative clinical assessment

Two pre-operative clinical scoring systems were used (clinical cases). Firstly, a standardized 21-point grading scheme for DLSS (19) was recorded by the attending board-certified veterinary neurologist. Secondly, the pet owner was invited to complete the canine brief pain inventory (CBPI) questionnaire (20), utilizing a quick-reference code to access an online form, that could be completed at home in private (Microsoft Forms, Microsoft, WA, USA). CBPI is a validated client-reported outcome measure for the treatment of osteoarthritis (21, 22) which has previously been used in dogs with lumbosacral pain (23, 24).

### 2.3 Pre-operative CT assessment

A planning CT scan of the lumbosacral region (Siemens Somatom Scope, 16 slice) was performed in all study subjects. Subjects were positioned in sternal recumbency with the pelvic limb joints flexed (sphinx position) and the lower back elevated using rolled towels positioned between the limbs.

The helical scan protocol encompassed images from the first lumbar vertebra to the first coccygeal vertebra. Acquisition parameters included a slice thickness of 0.75 mm, a pitch of 1, a matrix size of 512 x 512 and utilization of a bone kernel for image processing. *In-silico* planning was performed in commercially available DICOM (digital imaging and communications in medicine) viewing software (OsiriX MD Dicom Viewer Pixmeo Sarl^®^, version 13.0.1, Geneva, Switzerland) with 3D multi-planar reconstruction (MPR) perpendicular to the long axis of the vertebral column.

The MPR was aligned in a transverse plane to the level of the caudal aspect of the pedicle of L7 (at the cranial margin of the L7–S1 intervertebral foramen). This was used to assess vertebral and pelvic morphology and understand the relationship of the wing of the ilium to the intervertebral foramen, which was found to vary between each case. A 35–45° angled trajectory from the skin to the caudal pedicle was planned, and two measurements were recorded for later reference: the distance on the skin from mid-line, and the insertion depth from the skin to the caudal pedicle.

### 2.4 Equipment used

Standard surgical instruments included a no. 11 scalpel blade, surgical pen, ruler and needle holders.

**Figure 1:**
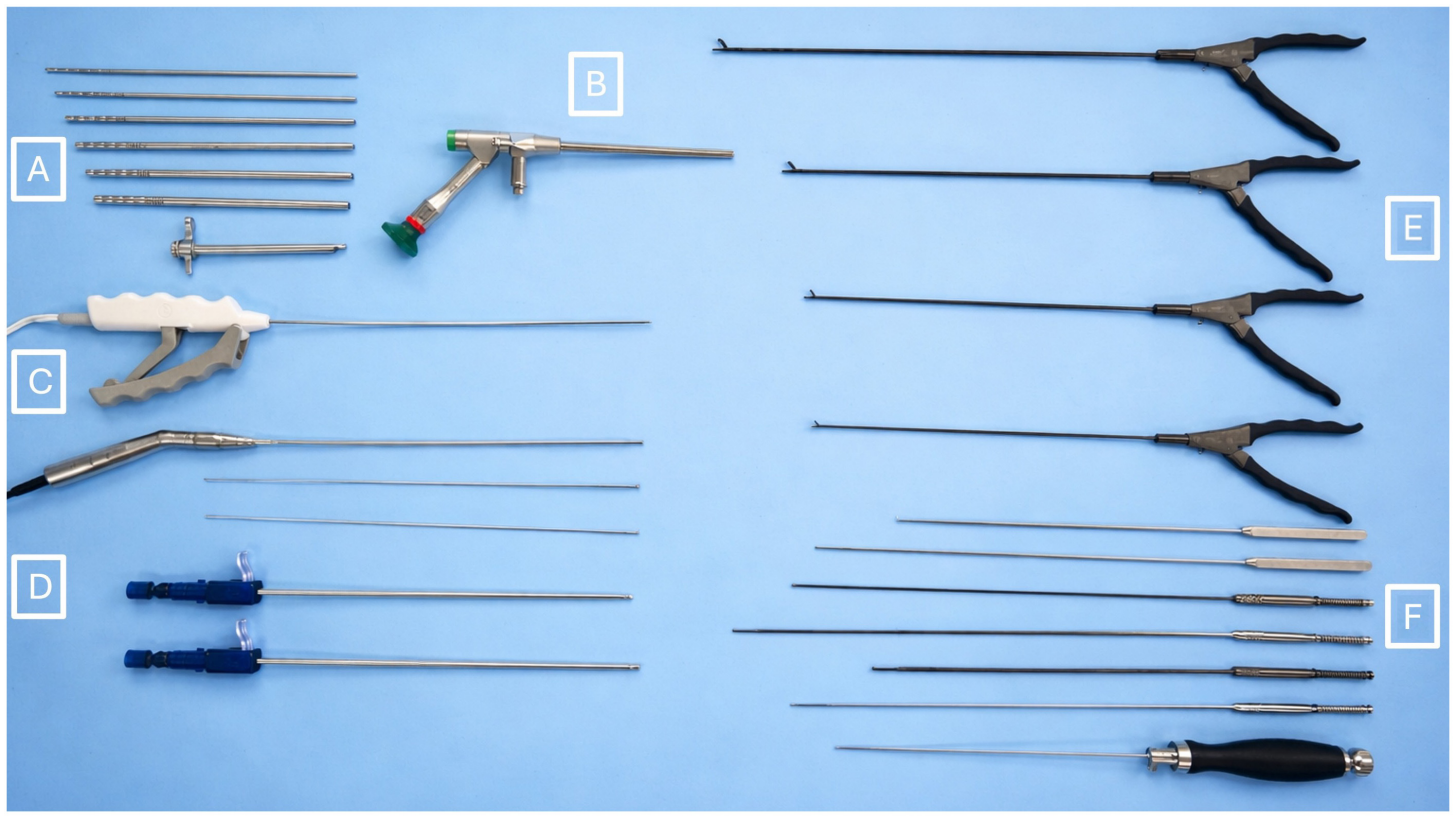
Surgical instrumentation for the lateral full-endoscopic lumbosacral foraminotomy procedure. **(A)** Serial muscle dilators and endosopic cannula. **(B)** 7 mm diameter, 130 mm long working-channel spine specific endoscope with 30° angled optics. **(C)** 28 cm long bipolar radiofrequency probe (FlexTip RF probe with WishBone^TM^ handle). **(D)** Endoscopic burrs for bone resection, from top to bottom; NSK Primado2 super-slim handpiece with 2 and 3 mm diameter round diamond burrs, Arthrex 3 mm diameter 330 mm long retractable oval and coarse diamond burrs. **(E)** Endoscopic graspers, from top to bottom; 3.5 mm Blakesley forceps, 3.5 and 2.5 mm cup forceps, 2 mm scissor punch. **(F)** Other instruments, from top to bottom; 2.5 mm hook probe, 2.5 mm blunt dissector, extrudable ball tip probe, 3.5, 2.5 and 1.7 mm Kerrison rongeurs, bone curette.

The following surgical instruments and equipment were supplied by Arthrex (Naples, FL, USA) for the duration of the study (Figure 1): Serial muscle dilators (diameters 2.5, 4.1, 5.1, 6 and 7 mm) and endoscopic cannula (8 mm diameter, 125 mm length, with elevator tip; A). A 7 mm diameter, 130 mm long working-channel spine specific endoscope with 30° angled optics (B). Cautery was provided with the Synergy^TM^ high frequency (4 MHz) electrosurgery unit and a 28 cm long bipolar radiofrequency probe (FlexTip RF probe with WishBone^TM^ handle; C). The Synergy^TM^ camera, light cable and UHD4 console unit and monitor were also used. Two spinal burr systems were utlised; 3 mm x 330 mm fluted and diamond barrel-shaped burrs with extendable hood (Arthrex, Naples, FL, USA) and 2.0/3.0 mm diamond and course diamond round-shaped endoscopic burrs (Primado 2, NSK Nakanishi Inc, Kanuma, Japan; D). Endoscopic graspers (E) included 3.5 mm Blakesley forceps, 3.5 and 2.5 mm cup forceps, 2 mm scissor punch. Other instruments included a 2.5 mm hook probe, 2.5 mm blunt dissector, extrudable ball tip probe, 3.5, 2.5 and 1.7 mm Kerrison rongeurs, and an endoscopic bone curette (F).

Fluid irrigation was managed using gravity (fluid bags elevated 0.5 m above the patient level) and gravity-fed tubing.

### 2.5 Surgical technique

The following technique was established and practiced in the cadaveric specimens, then performed in the clinical case series by the same surgeon (CD).

#### 2.5.1 Anesthetic protocol

Clinical cases were anesthetized using the same protocol. Premedication with methadone (0.2–0.3 mg/kg) and dexmedetomidine (3–5 mcg/kg), was administered intravenously or intramuscularly. General anesthesia was induced with propofol (2–4 mg/kg) to effect and maintained with isoflurane. An epidural injection of 1 mg/kg 0.5% bupivacaine and 0.1 mg/kg preservative free morphine was administered at L7-S1. Prophylactic antibiotics were given intravenously at induction (cefuroxime 20 mg/kg) and subsequently repeated every 90 min during the procedure. Perioperative analgesia was achieved with a continuous rate infusion of ketamine (5–10 mcg/kg/min). The short form of the Glasgow Composite Measure Pain Scale (CMPS-SF) was recorded every 4 h during hospitalization by independent observers (25).

#### 2.5.2 Patient position and room layout

Dogs were positioned as per the pre-procedural CT scan. The flexion of the hip joints and elevation of the caudal lumbar spine helped to flex the lumbosacrum, caudally withdraw the cranial articular processes of the sacrum from the caudodorsal aspect of the intervertebral foramen, and to caudodorsally deviate the iliac wings of the pelvis. This facilitates a dorsolateral approach to the lumbosacral intervertebral foramen that is perpendicular to the vertebral column with the patient in a stable position.

A radiolucent carbon fiber orthopedic operating table was used. The surgeon stands on the same side as the foramen to be operated. The C-arm image intensifier and scope tower are positioned opposite. The surgical instruments, electrocautery unit and electric burr system are positioned at the foot of the patient table. When a bilateral procedure is performed the c-arm and scope tower are maneuvered to the opposite side of the patient once the first foraminotomy is complete.

#### 2.5.3 Fluoroscopic targeting and cannula docking

**Figure 2:**
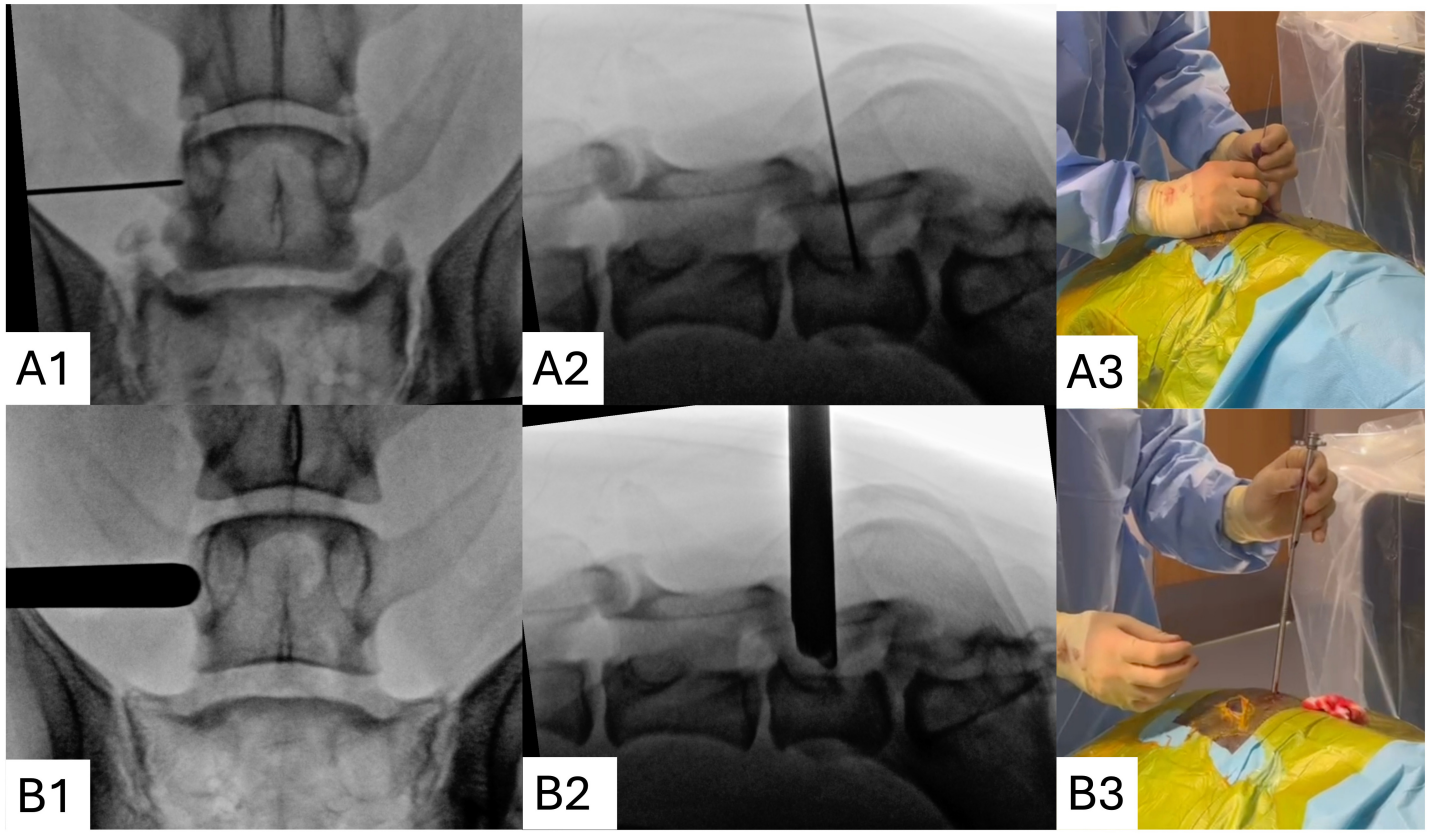
Docking the spinal endoscope cannula. A 1.1 mm k-wire is inserted through the incision, at a 35–45° angle from vertical, perpendicular to the L7 vertebral pedicle and is advanced until it contacts bone. Dorsal **(A1)** and lateral **(A2)** x-ray projections are used to confirm the correct location, at the base of the transverse process just cranial to the intervertebral foramen. Serial dilators are carefully introduced **(A3)** and after the surgeon has removed their hands from the surgical field x-rays projections are repeated **(B1, B2)** to check for deviation. Lastly, the endoscopic trochar is introduced **(B3)**.

Fluoroscopy was used to identify the location of the skin incision and to “dock” the endoscopic trochar adjacent to the intervertebral foramen (Figure 2).

Following aseptic patient preparation and draping, the C-arm image intensifier (Fujifilm FDR Cross) was orientated dorsoventral to the lumbosacrum, using its guidance laser. Local radiation safety rules concerning fluoroscopy were obeyed, including the use of personal and finger ring dosimeters. X-ray projection parameters varied according to the physical properties of each cadaver and clinical case (voltage 55–75 kV, collimation 14 cm, fluoroscopic mode at 25 frames per second). To limit radiation exposure, the continuous fluoroscopy function was limited to a fraction of a second with each exposure.

A sterile skin marker was used to mark the location of the initial skin incision. This mark was triangulated using a 0.9 mm diameter stainless steel k-wire held over the skin, so that dorsoventral x-ray projections could be used to identify the following landmarks: the sagittal mid-line (aligned to the dorsal spinous processes) and the transverse caudal most aspect of the L7 vertebral pedicle (just cranial to the cranial margin of the intervertebral foramen). The distance along the pedicular line had already been determined from the pre-operative scan, as previously described. An 8–10 mm stab incision was then made using a no. 11 blade, parasagittal to the vertebral column. The incision was extended deep to the subcutaneous tissue through the lumbodorsal fascia.

A 1.1 mm stainless steel k-wire was marked with the pre-determined insertion depth and was inserted through the para-spinal muscle using the 35–45° angled trajectory, until firm resistance from the vertebral pedicle was felt. The correct craniocaudal location of the pin was confirmed with a dorsoventral x-ray projection. The C-arm was then re-orientated perpendicular to the patient and a lateral x-ray projection was used to confirm the k-wire was docked onto the caudal pedicle at the level of the intervertebral foramen, close to the base of the vertebral canal. If the k-wire was incorrectly docked onto the lamina, it was re-orientated at a more acute insertion trajectory and the process repeated until the docking location was correct. The muscle dilators were sequentially inserted over the k-wire and lastly the 8 mm diameter endoscopic trochar was inserted, before the dilators removal. The elevator tip of the trochar was orientated cranially. Intermittent dorsoventral x-ray projections were made during the process to confirm the final “docked” location of the trochar (Figure 2).

#### 2.5.4 Soft-tissue management and landmark identification

With the C-arm still in position, the endoscope was introduced into the trochar with active fluid irrigation. The remaining muscle at the base of the trochar is resected using a mixture of bipolar electrocautery, Blakesley and cup forceps. The bipolar cautery tip could be extruded at an angle toward the periphery of the endoscopic view to manage any minor soft-tissue bleeding. If significant soft-tissue bleeding was encountered, a small piece of wet-stable collagen haemostat (Lyostypt? BBraun, Tuttlingen, Germany) was introduced into the working channel and was compressed using the nerve hook. The irrigation pressure could be temporarily increased by occluding the fluid-outflow port of the endoscope.

**Figure 3:**
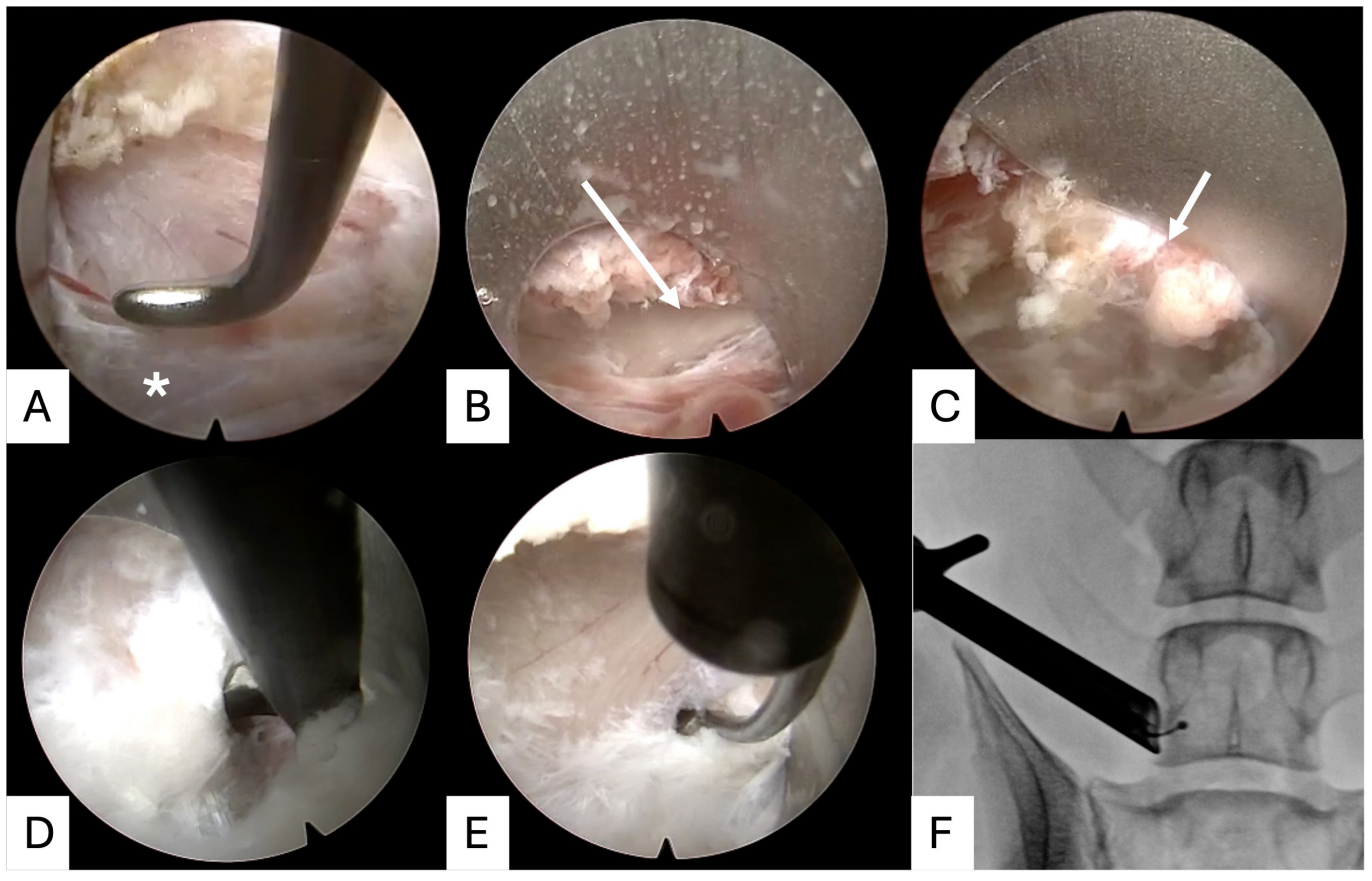
Determination of anatomic landmarks (cranial is to the left of all images). Following initial soft-tissue clearance, the base of the transverse process can be palpated (asterisk, **A**). The trochar can be used to clear muscle attachments from the caudal articular process of L7 (arrowed, **B**) until the trochar sits on top of the L7-S1 facet joint (arrowed, **C**). The foraminal ligament is palpated and a small window can be made with a scissor-punch rongeur **(D)** before the dorsal aspect of the foramen is gently palpated with a ball-tipped probe **(E)**, the position of which can be checked in relation to the lateral recess with a dorsoventral x-ray **(F)**.

Several anatomic landmarks could be directly visualized and/or palpated with the elevator tip of the trochar, which serves as the surgeons soft-tissue retractor. Palpable landmarks included the base of the transverse process of L7, the caudal articular process of L7 with the dorsal border of the intervertebral foramen, and the transforaminal ligament of L7–S1 (Figure 3). The bipolar cautery could be used to palpate and thin out this ligament. If necessary, a scissor-punch could be used to create a small opening into the foramen. A ball-tipped probe could be extruded into the foramen to palpate the caudal aspect of the pedicle and fluoroscopically confirm the trajectory of the lateral recess prior to bone resection (Figure 3).

#### 2.5.5 Endoscopic bone resection

**Figure 4:**
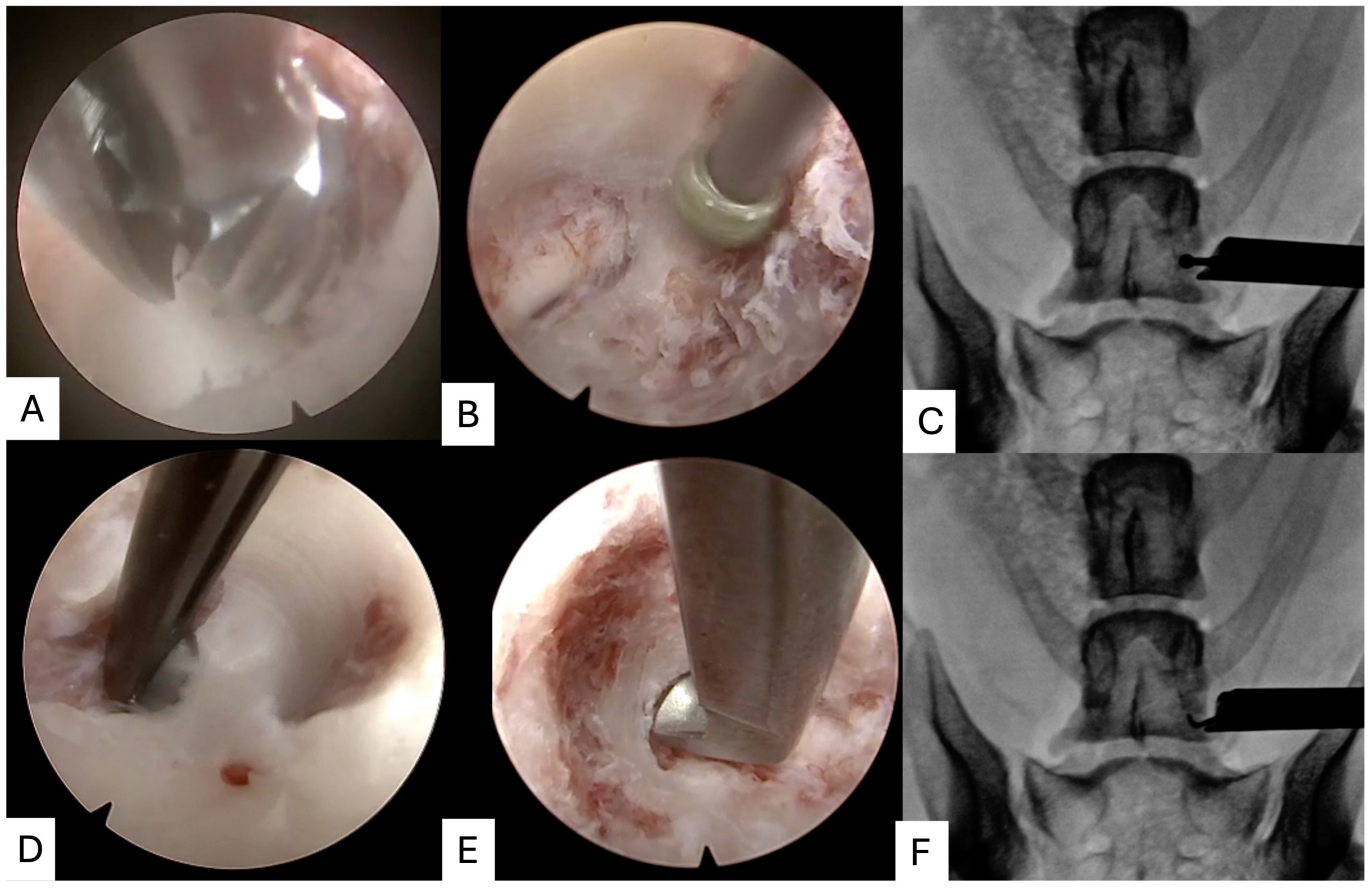
Endoscopic bone resection. The foraminotomy can be started with a 3 mm diameter fluted barrel burr (**A**; cranial to right) and continued with 2 and 3 mm diamond burrs (**B**; cranial to right). Dorsoventral x-ray projections can be used to check progress through the pedicle/proximity to the inner cortical bone **(C)**. The foraminotomy can be extended using 2.5 mm (**D**; cranial to right) and 1.7 mm (**E**; cranial to left) Kerrison rongeurs. Dorsoventral x-ray can also be used to check the craniocaudal extent of the foraminotomy **(F)**.

The extent of bone resection for the foraminotomy was performed as previously described (2). Initially, the fluted barrel-shaped burr or course diamond burr was used to resect the bone of the ventrolateral pedicle craniodorsal to the predicted exit of the L7 spinal nerve root. If necessary, the distance to the inner cortical bone could be assessed with a dorsoventral x-ray (Figure 4). Once the inner cortical bone was thinned, the L7 spinal nerve root could be visualized with the periosteum intact. A 2 mm diameter fine diamond burr and 45° angled Kerrison rongeurs were used to complete the bone removal (Figure 4). Cancellous bone bleeding was minimal and was managed with fluid irrigation only.

#### 2.5.6 Completion of foraminotomy and determination of procedure end point

**Figure 5:**
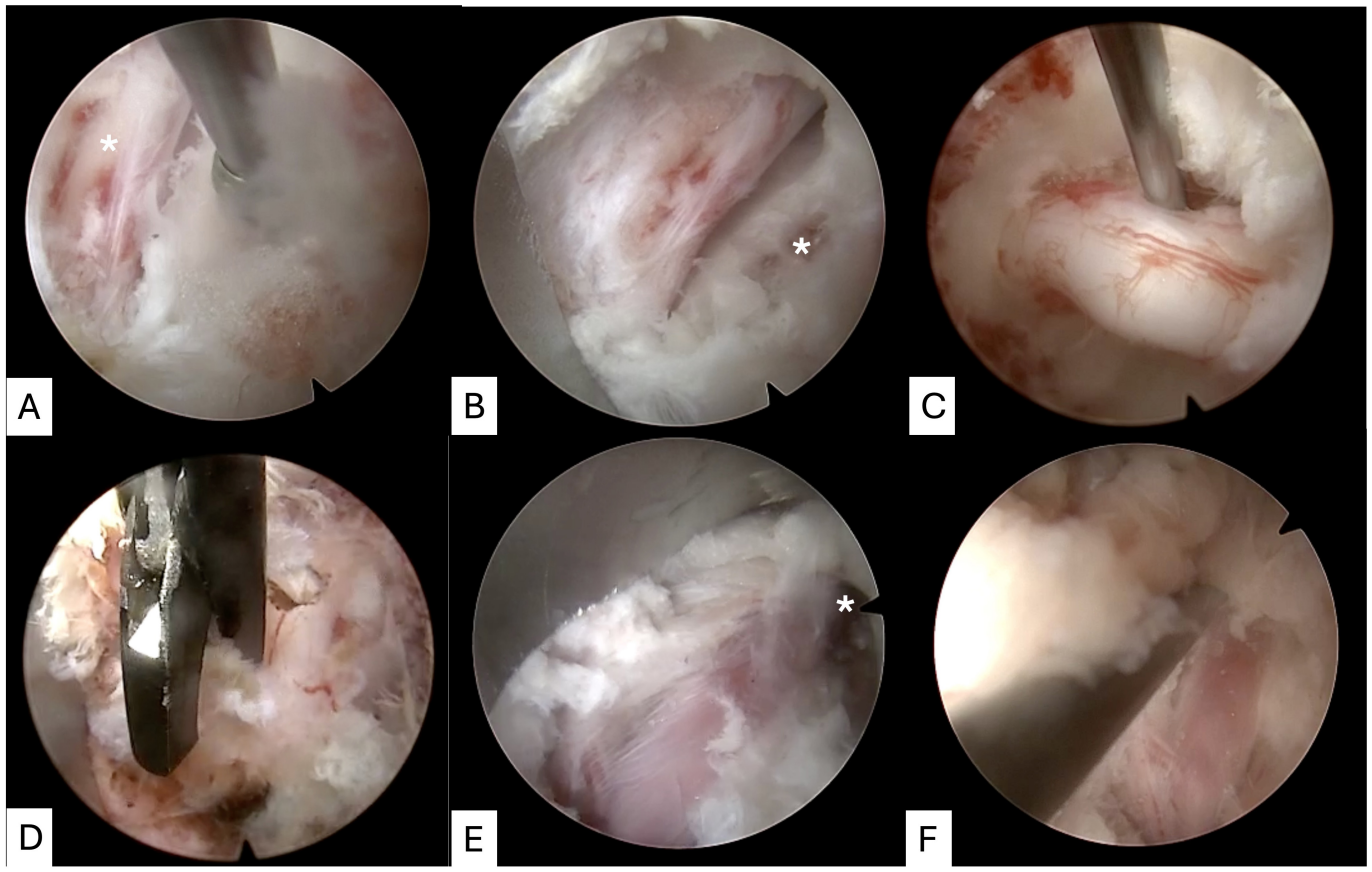
Completion of foraminotomy and determination of procedure end-point. The endoscope is rotated 90° to the existing L7 spinal nerve root (asterisk, **A**; cranial to right), such that the working channel for introduction of the burr can now resect the remaining “lip” of pedicle (asterisk, **B**; cranial to right). A probe can then be used to check the nerve root can be exteriorised (**C**; cranial to left). Fibrous adhesions on then distal portion of the nerve root can be gently resected using a scissor punch (**D**; cranial to right). The endoscope can be rotated 180° to the surgeon to inspect (asterisk, **E**; cranial to bottom and lateral to right) and palpate **(F)** the post-foraminal nerve root.

Care was taken to remove the ventrolateral aspect of the pedicle which required some removal of bone at the dorsolateral aspect of the vertebral body. Turning the endoscope 90° was helpful in changing the orientation of the working channel to avoid iatrogenic injury to the spinal nerve root (Figure 5). The foraminotomy was considered complete when the dorsal root ganglion and the spinal nerve root proximal and distal to this structure could be visualized, and a nerve hook could be used to temporarily exteriorize the nerve root from the foramen. Finally, the endoscope and trochar could be rotated 180° to visualize the post-foraminal nerve root and assess for any soft-tissue adhesions (Figure 5). The endoscope and trochar were withdrawn from wound and the skin was closed with a single cruciate pattern suture. A post-operative CT scan was performed to determine procedure efficacy.

### 2.6 Outcome measures

#### 2.6.1 Intra-operative assessments

The following quantitative and qualitative assessments were made from intra-operative observations:

- Surgical time:
  This was determined as the number of minutes taken from the start of the fluoroscopic targeting to the determination of the procedure end-point, as described above, for each operated foramen.
- Landmark visualization quality:
  Five bony and soft-tissue landmarks were identified and recorded during each procedure; the base of the transverse process of L7, the base of the caudal articular process of L7, the transforaminal ligament L7–S1, the spinal nerve root of L7 and the epidural space of the vertebral canal caudodorsal to the spinal nerve root of L7. The quality of visualization was assigned a score as follows: Good (all five identified), fair (three or four of five identified) or poor (one or two identified).
- Iatrogenic nerve root injury:
  The safety of the procedure was determined by recording observations of dural tears, partial or complete nerve root laceration.
- Incision length:
  The incision length was determined by recording the length of the final skin incision using a digital caliper (rounding up to the nearest mm) and whether it was necessary to convert to an open procedure to reach the described procedure endpoint.

#### 2.6.2 Post-operative assessments

The following post-operative clinical and radiologic parameters were assessed:

- Neuroforaminal volume enlargement:
  For all study subjects the efficacy of the procedure was evaluated using pre- and post- operative CT scans by an independent observer (JR). The volume (mm^3^) of the L7–S1 intervertebral foramen was calculated using an *in-silico* segmentation and measurement methodology as previously described (26) but performed with open-access software (3D Slicer software; Surgical Planning Lab, Harvard Medical School, Harvard University, Boston, MA, USA, http://www.slicer.org, accessed on 3 September 2025). The difference in the two volumes was used to calculate foraminal enlargement.
- Clinical hospitalization parameters (clinical case series):
  Electronic hospitalization records were retrospectively reviewed for the following: Firstly, the hospitalization period was recorded as < 24 h where discharge occurred the same day as the surgical procedure, 24 h where discharge occurred the following morning, or >24 h when discharge occurred thereafter. Secondly, the use of injectable opioids (methadone) during post-operative hospitalization was recorded as necessary or not necessary; in our institution, a CMPS-SF of >5 out of 24 prompts the use of additional analgesia.
- Medium-term follow up (clinical case series):
  Clinician (DLSS score) and carer-reported (CBPI) outcome measures were determined at 6 months post-operative during follow up in-person consultation.

### 2.7 Statistical evaluation

Neuroforaminal volume data analysis was performed using GraphPad Prism (version 10.0 for macOS, GraphPad Software, Boston, MA, USA). The Shapiro–Wilk test was used to assess the normality of this continuous variable, and variables were expressed as median ± range. The Wilcoxon matched pairs signed rank test was used to compare the median neuroforaminal volumes pre- vs. post-procedure.

## 3 Results

### 3.1 Study subjects

The *ex-vivo* portion of the study consisted of two large breed dog cadavers, both golden retrievers, weighing 32 and 35 kg. In both cadavers, LELF was successfully performed bilaterally, prior to clinical case recruitment.

Three clinical cases were recruited: a 36 kg German Shephard dog, a 27 kg cross breed and a 25 kg English Springer Spaniel (Table 1). The initial diagnosis had been made 6, 8 and 36 weeks prior to surgery, respectively (case 3 had CT repeated 8 weeks prior to surgery to confirm the diagnosis and exclude other differentials). Following diagnosis, exercise had been restricted to controlled lead walks of 10–15 min duration. Medical therapy was administered for at least 6 weeks and consisted of a non-steroidal anti-inflammatory drug (meloxicam 0.1 mg/kg once daily in case 1 and 3, robenacoxib 1 mg/kg once daily in case 2), gabapentin (10 mg/kg three times daily, all cases), memantine (0.5 mg/kg once daily, case 3) and paracetamol (15 mg/kg three times daily, cases 2 and 3). Following diagnosis, case 3 had been treated with a single injection of 1 mg/kg methylprednisolone acetate into the dorsal epidural space at L7–S1. All three dogs suffered relapses of clinical signs following conservative medical management. Caregivers reported no improvement in gait and postural abnormalities, with behavioral changes including poor demeanor and increased reactivity to other dogs and on handling, prompting the decision to proceed with surgery.

**Table 1:** Signalment, pre- and post-operative clinical assessments for clinical cases.

Clinical case #	Breed	Age (years)	Gender	Weight (kg)	Foraminal stenosis	DLSS score (pre)	CBPI score (pre)	Hospital period	Opioid administration	DLSS score (follow-up)	CBPI score (follow-up)
1	GSD	5	F	36	Right	12	88	< 24 h	Not necessary	21	0
2	Cross Breed	9	MN	27	Bilateral	16	73	< 24 h	Not necessary	19	15
3	ESS	11	FN	25	Bilateral	15	68	24 h	Not necessary	19	8

Key: GSD, German Shepherd dog; ESS, English Springer Spaniel.

On examination, case 1 had moderate right pelvic limb lameness when walking and intermittent non-weight bearing when standing, consistent with a nerve root signature. There was mild muscle atrophy in the affected limb. There was resistance to right hip extension, interpreted as probable pain. Cases 2 and 3 suffered mild bilateral pelvic limb lameness, with difficulty jumping. On neurologic examination, all three cases had normal postural reactions, segmental spinal reflexes and normal tail movement. All three cases had reaction on firm dorsal pressure of the lumbosacrum and failure of the lordosis test, consistent with pain. The remainder of orthopedic and neurologic examinations were unremarkable.

**Figure 6:**
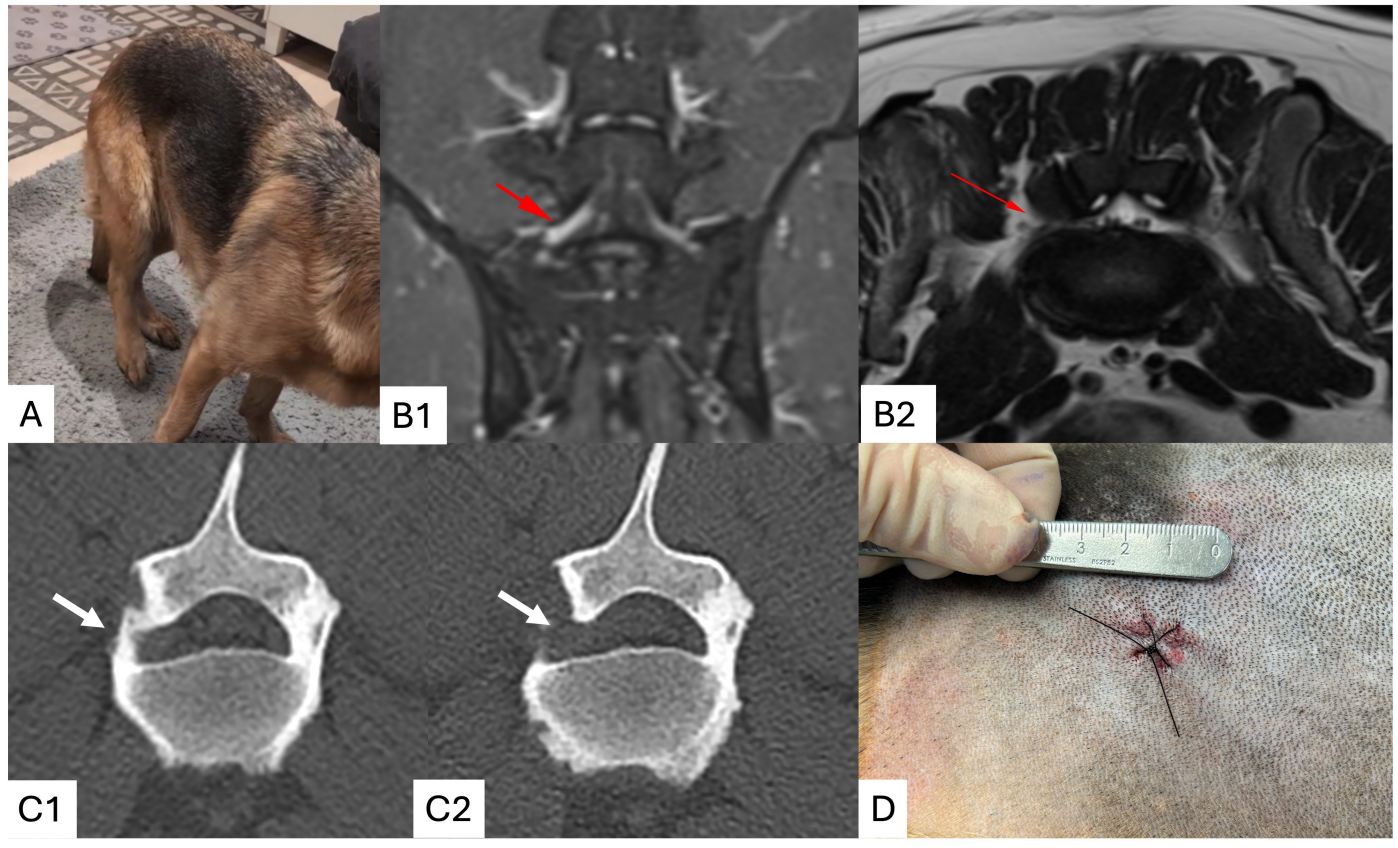
Clinical case 1. An intermittent non-weight bearing right pelvic limb lameness was present, suggesting a “root signature” **(A)**. Dorsal STIR **(B1)** and transverse T2-weighted **(B2)** MRI scans of the lumbosacrum demonstrate foraminal stenosis and increased signal in the L7 spinal nerve root (red arrows). Pre **(C1)** and post **(C2)** CT scans in a bone window at the level of the caudal vertebral body of L7 demonstrate resection of the ventrolateral pedicle (arrows). The surgical wound is less than 10 mm in length **(D)**.

Written informed consent was obtained including agreement to LELF and the potential need to convert to an open technique if needed. Case 1 was previously diagnosed with unilateral LSFS and so underwent unilateral LELF (Figure 6). Cases 2 and 3 were previously diagnosed with bilateral LSFS and underwent bilateral LELF. In all instances, LSFS was determined to occur in the foraminal middle and exit zones and lateral foraminotomy was considered an appropriate surgical therapy (3, 5).

### 3.2 Pre-operative clinical assessment scores

DLSS scores were 12, 16, and 15, for cases 1–3 respectively. The pet owners scored their CBPI as 88, 73, and 68 respectively (Table 1). Quality of life was reported as poor (cases 1 and 3) or fair (case 2).

### 3.3 Intra-operative assessments

Intra-operative assessments are reported in Table 2. In total, nine foramina were operated (four cadaveric, five clinical). It was never necessary to convert to an open procedure and therefore the incision lengths varied between 8 and 10 mm, as originally planned. The median surgical time was 95 min (range 91–110 min) per foramen. For all foramina, all necessary anatomic landmarks could be visualized (quality recorded as good). No dural tears, partial or complete nerve root injuries were seen.

**Table 2:** Intra- and post-operative assessments for all cases.

Case #	Foramen	Surgical time (min)	Vizualisation quality	Incision length (mm)	Pre-operative volume (mm^3^)	Post-operative volume (mm^3^)	Foraminal enlargement (mm^3^)
Cadaver 1	Left	110	Good	10	288.88	605.57	316.69
	Right	103	Good	9	206.78	594.32	387.54
Cadaver 2	Left	94	Good	9	463.02	665.38	202.36
	Right	92	Good	10	482.32	722.75	240.43
Clinical 1	Right	95	Good	8	850.47	1,402.69	552.22
Clinical 2	Left	98	Good	9	398.30	674.86	276.56
	Right	109	Good	8	315.22	534.16	218.76
Clinical 3	Left	91	Good	10	221.71	511.47	289.76
	Right	93	Good	8	279.93	520.97	241.04

### 3.4 Post-operative assessments

**Figure 7:**
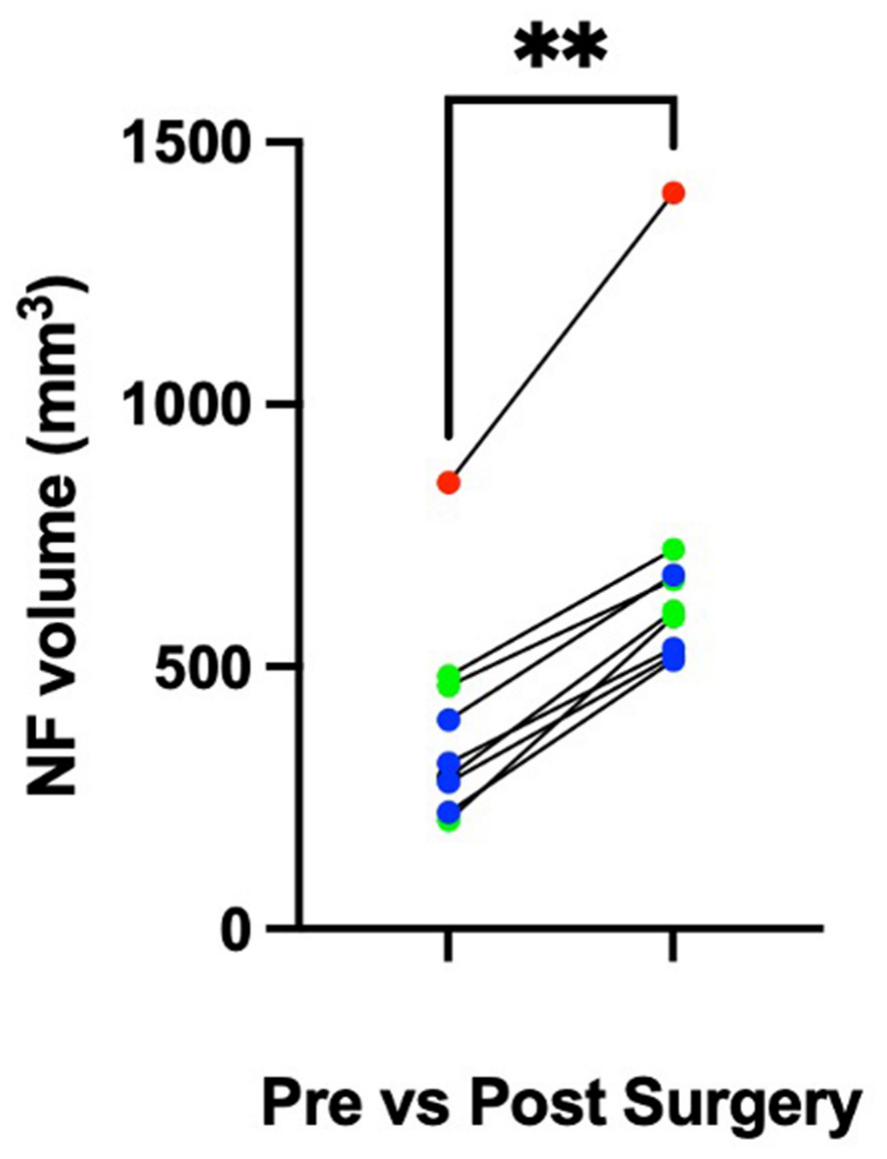
Neuroforaminal enlargement. Pre- vs. post-operative neuroforaminal volumes (mm^3^) of cadavers (blue), clinical cases 1 (red), 2 and 3 (green). **p < 0.05.

Post-operative assessments for clinical cases are reported in Table 1. There was a significant (*P* = 0.004) increase in median foraminal volume pre- (315.2 mm^3^, range 206.8–850.5 mm^3^) vs. post- (605.6 mm^3^, range 511.5–1,403 mm^3^) operative (Figure 7). For cases 1 and 2, dogs were discharged the morning after surgery (< 24 h) whereas case 3 was discharged just over 24 h later, but this related to the caregivers availability to collect the dog. In all cases, pain-scores were not sufficient to prompt the administration of an injectable opioid medication.

Following discharge, exercise was restricted to 10 min of controlled lead walking, for 2 weeks. After this time, no specific restrictions were recommended. Oral medications administered prior to surgery were continued during this 2-week period, after which NSAIDS and paracetamol were withdrawn. Treatment with gabapentin (10 mg/kg three times daily) continued during the follow-up period.

All three cases improved clinically; case 1 enjoyed a complete recovery (DLSS score 21 and CBPI score 0), cases 2 and 3 scored 19/15 and 19/8 respectively. The residual clinical signs were mild, including persisting resentment of firm dorsal lumbosacral palpation (both cases), hesitation to jump (both cases) and transient stiffness after standing (case 2), despite a return to normal exercise levels and client-reported assessment of quality of life on CBPI as excellent.

## 4 Discussion

In this study we successfully developed and described a technique for LELF in dog cadavers and in three clinical cases of LSFS. We therefore accepted our hypothesis that LELF would be feasible and given the small incision and muscle-sparing approach, the procedures were minimally invasive. Significant enlargement of the intervertebral foramen was determined in all study subjects. The exact volume of bone resection and foraminal enlargement required for a successful clinical outcome is yet to be determined in dogs, however, removal of the lip of bone at the ventrolateral pedicle is desirable (3).

The closest analog to LELF in human neurosurgery is the transforaminal approach for full-endoscopic discectomy or foraminotomy (18, 27). The main difference to LELF is the ability to bypass the bone and exiting nerve root to dock the spinal endoscope directly onto the pathologic disc. Given that dogs have a relatively small intervertebral foramen and large spinal nerve root, it was necessary for us to develop an initial docking technique cranial to the exiting nerve root and foramen, with bone resection taking place prior to visualization of the spinal nerve root. Despite this disadvantage, we did not visualize dural or spinal nerve root injury and we did not induce neurologic deficits or lameness in the clinical cases. Therefore, we also accepted our hypothesis that LELF was a safe procedure; however, these risks will remain procedural considerations given their incidence in human endoscopic spinal surgery (28) and the limited case numbers presented here.

The medium-term outcome of the clinical cases was good, considering the improvements in surgeon (DLSS score) and pet carer (CBPI score) reported outcomes, including restoration of excellent qualities of life. This is in agreement with previous reports of medium-term outcome assessment for open lateral foraminotomy procedures (3, 29, 30). As DLSS and LSFS are progressive diseases, the potential for clinical relapse exists (29), and would be better determined over a longer period of follow-up. Potential reasons for relapse of clinical signs might include the development of contralateral foraminal stenosis (29) or the reformation of bone resulting in recurrent stenosis, which might limit the long-term effectiveness of decompression (30). In the three clinical cases presented here, foraminal stenosis was determined to occur in the foraminal middle and exit zones, making them good candidates for lateral foraminotomy (3). Failure to improve following lateral foraminotomy has been reported in a small number of working dogs leading to a subsequent vertebral fixation procedure in one case (30). Future long-term study is required to determine whether LELF is similarly prone to bony reformation and relapse, or failure to initially improve. However, our description of the LELF procedure preserves sufficient bone stock in the vertebral pedicle to accept an orthopedic implant, if a fixation procedure is required.

The median time for each foraminotomy was 95 min, owing to the novelty of the procedure and lack of familiarity with the endoscopic equipment. It is well recognized in human neurosurgery for endoscopic spinal surgery to be associated with a steep learning curve, with the adoption of a uniportal technique in one study leading to median operative time decreasing by half over the first 50 patients (31). Unilateral biportal endoscopic (UBE) surgery is an alternative technique described for minimally invasive lumbar discectomy, utilizing standard arthroscopic equipment introduced through a separate incision (portal) to the camera (32). In comparison to UBE, uniportal full-endoscopic procedures have a steeper and longer learning curve (33). UBE has recently been described as a minimally invasive technique for lateral foraminotomy in dog cadavers (15). The primary surgeon in the current study (CD) is a neurology diplomate with no previous experience in triangulating arthroscopic equipment. Whether UBE would have a shallower learning curve than LELF for veterinary surgeons experienced in arthroscopy is currently unknown.

As previously mentioned, the main limitations of this study are the limited number of clinical cases and the medium-term clinical follow up period. This limits our ability to draw conclusions about ideal case selection, operative risk, degree of ideal foraminal enlargement and long-term success rates. The low pain scores recorded during hospitalization and the ability to discharge patients within 24 h of the procedure may be, at least in part, attributed to the local anesthetic epidural block.

Another limitation of LELF is the necessity for specific instrumentation and surgical facilities, which could pose logistical and financial challenges to widespread adoption of this technique.

Other less invasive image-guided therapies such as foraminal steroid injections, with or without adjunctive pulsed radiofrequency, have recently been described (24) and could be considered in selected cases of LSFS prior to considering LELF.

In conclusion, the LELF technique was successfully established. LELF offers an effective and minimally invasive option for lateral foraminotomy, which improved clinical signs in three dogs with LSFS.

## Data Availability

The raw data supporting the conclusions of this article will be made available by the authors, without undue reservation.
